# Supplementary material for: Influence of the Cholinergic System on the Pathogenesis of Glioblastoma: Impact of the Neutrophil Granulocytes
Source: Int J Mol Sci. 2025 Dec 27;27(1):321. doi: 10.3390/ijms27010321 (PMC12785807; doi:10.3390/ijms27010321)
Supplement: Supplementary file 1 [file ijms-27-00321-s001.zip › Suplemmentary Figure S1.pdf]

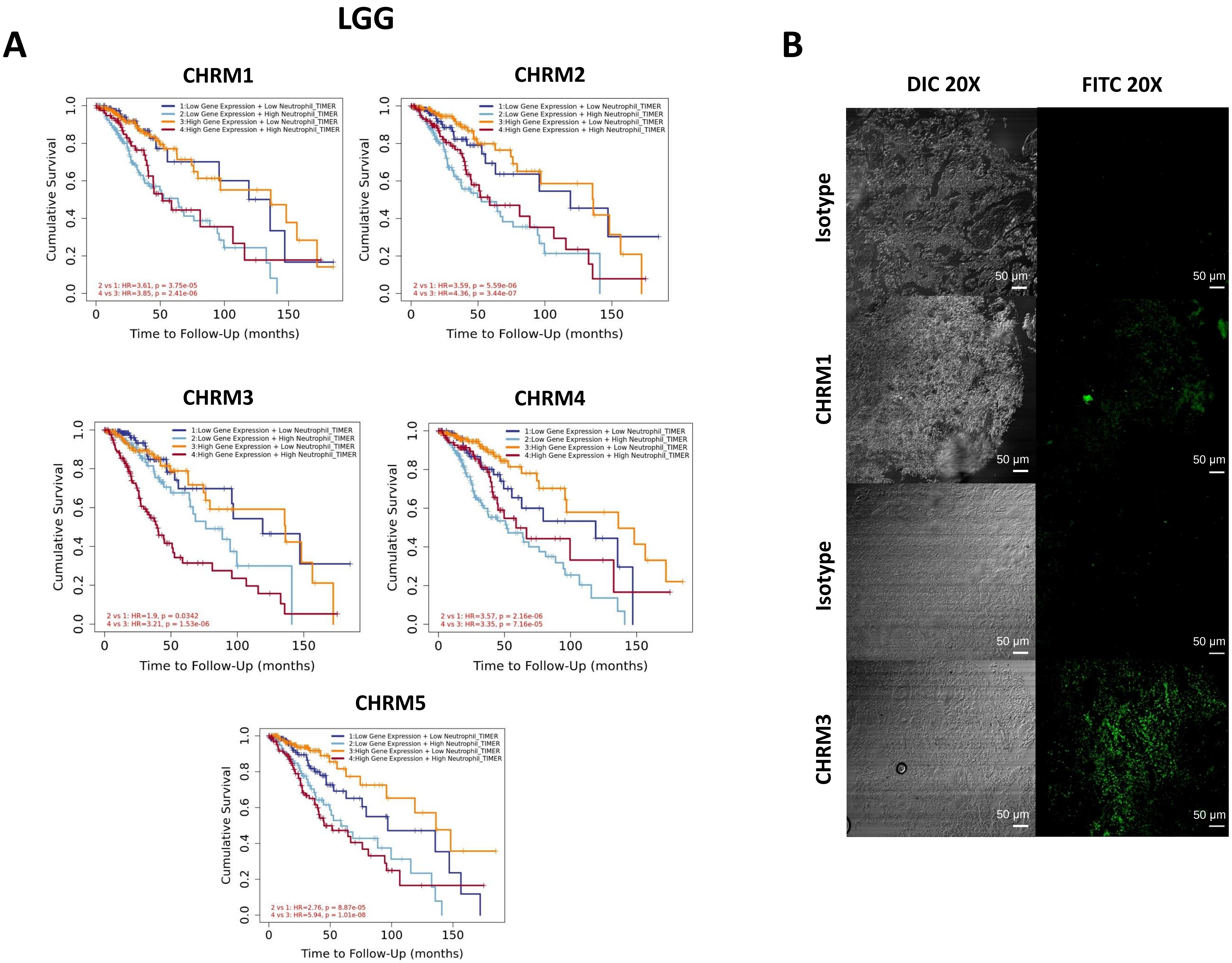
LGG
A
CHRM1
1:Low Gene Expression + Low Neutrophil_TIMER
2:Low Gene Expression + High Neutrophil_TIMER
3:High Gene Expression + Low Neutrophil_TIMER
4:High Gene Expression + High Neutrophil_TIMER
2 vs 1: HR=3.61, p = 3.75e-05
4 vs 3: HR=3.85, p = 2.41e-06
Cumulative Survival
Time to Follow-Up (months)
CHRM2
1:Low Gene Expression + Low Neutrophil_TIMER
2:Low Gene Expression + High Neutrophil_TIMER
3:High Gene Expression + Low Neutrophil_TIMER
4:High Gene Expression + High Neutrophil_TIMER
2 vs 1: HR=3.59, p = 5.59e-06
4 vs 3: HR=4.36, p = 3.44e-07
Cumulative Survival
Time to Follow-Up (months)
CHRM3
1:Low Gene Expression + Low Neutrophil_TIMER
2:Low Gene Expression + High Neutrophil_TIMER
3:High Gene Expression + Low Neutrophil_TIMER
4:High Gene Expression + High Neutrophil_TIMER
2 vs 1: HR=1.9, p = 0.0342
4 vs 3: HR=3.21, p = 1.53e-06
Cumulative Survival
Time to Follow-Up (months)
CHRM4
1:Low Gene Expression + Low Neutrophil_TIMER
2:Low Gene Expression + High Neutrophil_TIMER
3:High Gene Expression + Low Neutrophil_TIMER
4:High Gene Expression + High Neutrophil_TIMER
2 vs 1: HR=3.57, p = 2.16e-06
4 vs 3: HR=3.35, p = 7.16e-05
Cumulative Survival
Time to Follow-Up (months)
CHRM5
1:Low Gene Expression + Low Neutrophil_TIMER
2:Low Gene Expression + High Neutrophil_TIMER
3:High Gene Expression + Low Neutrophil_TIMER
4:High Gene Expression + High Neutrophil_TIMER
2 vs 1: HR=2.76, p = 8.87e-05
4 vs 3: HR=5.94, p = 1.01e-08
Cumulative Survival
Time to Follow-Up (months)
B
DIC 20X
FITC 20X
Isotype
CHRM1
Isotype
CHRM3
50 µm

Figure S1. Relevance of CHRM expression in LGG and GBM patients. (A) Meta-analysis of TCGA patient gene expression databases evaluating CHRM1, CHRM2, CHRM3, CHRM4, and CHRM5 expression in LGG samples and their correlation with neutrophil infiltrate and overall survival. Analysis performed with TIMER2.0 (Tumor Immune Estimation Resource; http://timer.cistrome.org/ (accessed on May 2025)). LGG = 516. Spearman correlation, $p < 0.05$. (B) CHRM 1 and CHRM3 expression in GBM-b samples evaluated by immunostaining (20× magnification). Representative experiment from six patients.
