# Supplementary material for: Influence of the Cholinergic System on the Pathogenesis of Glioblastoma: Impact of the Neutrophil Granulocytes
Source: Int J Mol Sci. 2025 Dec 27;27(1):321. doi: 10.3390/ijms27010321 (PMC12785807; doi:10.3390/ijms27010321)
Supplement: Supplementary file 1 [file ijms-27-00321-s001.zip › Suplemmentary Figure S2.pdf]

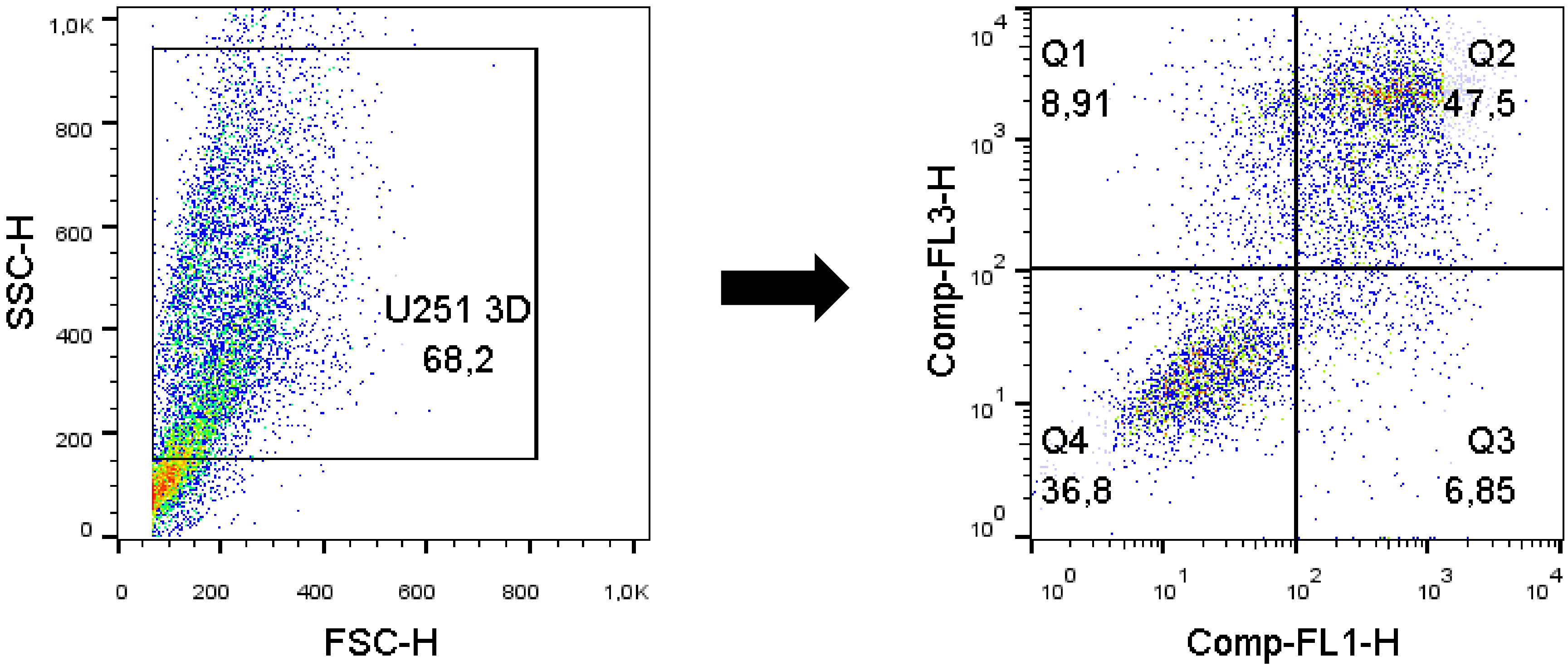

Figure S2. Gating strategy for apoptosis analysis by phosphatidylserine exposure, detected by annexin V and propidium iodide stanning in the U251 tumor cell line.
